# Tight Junctions of the Neurovascular Unit

**DOI:** 10.3389/fnmol.2021.752781

**Published:** 2021-11-19

**Authors:** Natalie Hudson, Matthew Campbell

**Affiliations:** Trinity College Dublin, Smurfit Institute of Genetics, Dublin, Ireland

**Keywords:** tight junction, neurovasculature, endothelial cells, blood brain barrier, inner blood-retinal barrier

## Abstract

The homeostatic balance of the brain and retina is maintained by the presence of the blood-brain and inner blood-retinal barrier (BBB/iBRB, respectively) which are highly specialized barriers. Endothelial cells forming the lining of these blood vessels are interconnected by the presence of tight junctions which form the BBB and iBRB. These tight junctions, formed of numerous interacting proteins, enable the entry of molecules into neural tissues while restricting the entry of harmful material such as anaphylatoxins, bacteria and viruses. If the tight junction complex becomes dysregulated due to changes in expression levels of one or more of the components, this can have detrimental effects leading to brain and retinal pathology.

## Introduction

Vascular heterogeneity is essential for the diverse functions and roles arising across the vascular tree; and is of particularly great importance in the brain and retina. The microvasculature of the brain and the retina differs vastly to other vascular beds due to the presence of the blood-brain (BBB) or inner blood-retinal barrier (iBRB) which are formed from endothelial cells that interconnect via highly specialized and enriched tight junctions that act as selective barriers (Abbott et al., 2006; Hudson and Campbell, 2019). The tight junctions help to regulate the entry of molecules and ions, from the blood into the tissue while restricting entry of potential harmful blood-borne components, including immune cells and pathogens (Abbott et al., 2006). In addition to the endothelial cells, the BBB and iBRB requires the presence of astrocytes, pericytes, microglia, Müller cells, and the basement membrane to help facilitate the barrier properties that are intrinsic within the brain and retina. The microenvironment needs to be stringently controlled to maintain homeostatic conditions, as dysfunction of junctional components can lead to numerous brain and retinal pathologies (as shown in Table 1).

**TABLE 1:** Contribution of tight junction components to disease pathology.

**Tight junction component**	**Disease pathology**
Claudin-5	• EAE/MS: claudin-5 loss and remodeling during leukocyte transmigration • RPE atrophy observed in response to claudin-5 downregulation (animal model of dry AMD) • Decreased claudin-5 levels detected in post-mortem brains of individuals diagnosed with Schizophrenia • Decreased claudin-5 levels found in Epilepsy • Stroke • Cold-induced model of traumatic brain injury (TBI) found decreased claudin-5 levels reduced edema and accelerated recovery • Repetitive mild TBI found decreased claudin-5 levels in association with deposition of hyperphosphorylated tau leading to BBB dysfunction • Alzheimer’s disease found increased amyloid-β clearance into blood when claudin-5 and occluding down-regulated. • Claudin-5 knockdown exacerbates social defeat model of depression • Claudin-5 mislocalization and increased expression in oxygen induced retinopathy (OIR) model
Claudin-1	• Increased expression in stroke • Decreased expression in Glioblastoma Multiforme • Expression of claudin-1 reduces vascular leakage in model of EAE
Claudin-3	• Decreased expression observed in EAE and Glioblastoma Multiforme
Occludin	• Lower occludin levels observed in Multiple Sclerosis • VEGF mediated phosphorylation of occludin in Diabetic Retinopathy leads to dysfunctional iBRB • Alzheimer’s disease found increased amyloid-β clearance into blood when claudin-5 and occludin down-regulated
Zonula Occludens (ZO-1)	• In Multiple Sclerosis lesions ZO-1 expression reduced leading to junctional instability
LSR	• Downregulated in EAE/middle cerebral artery occlusion leading to junctional instability
JAM-A	• Loss of JAM-A leads to increased neutrophil transmigration • Increased JAM-A expression correlates with increased monocyte migration in HIV-infected individuals
JAM-C	• Down-regulation of JAM-C inhibits wet AMD patient macrophage adhesion to endothelial cells

## Composition of the Neurovascular Unit

**FIGURE 1:**
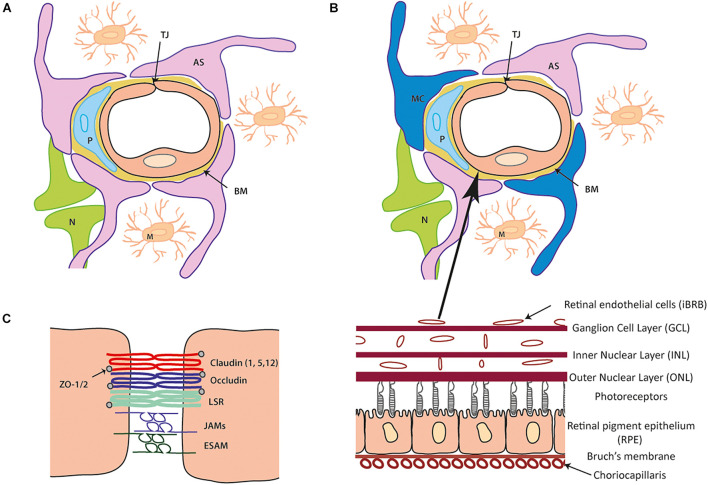
Cellular and tight junction (TJ) protein composition of the blood- brain barrier (BBB) and inner blood-retinal barrier (iBRB). **(A)** Schematic of the blood brain barrier (BBB) neurovascular unit (NVU). A single endothelial cell (EC) forms the lumen of the blood vessels surrounded by a pericyte (P) and the basement membrane (BM) containing laminins, nidogens, collagen IV and heparin sulfate proteoglycans. Astrocytes (AS) end-feet ensheath the cell complex with neurone (N) and microglia (M) present in the microenvironment. **(B)** The iBRB is similar in composition to the BBB (as seen in **A**) although pericytes (P) are at a ratio of 1:1 with endothelial cells (EC) and Muller cell (MC) processes wrap around the blood vessels along with the astrocytes (AS). The iBRB is found in the retina from the ganglion cell layer (GCL) to the outer nuclear layer (ONL). **(C)** Schematic of tight junction proteins expressed that join the same endothelial cell or adjacent endothelial cells to one another. Claudin-5 is expressed most abundantly with contribution from claudin 1 and 12 (other family members shown to be expressed in other NVU cells). The TAMPs (occludin, tricelllin) and LSR along with JAM family members (**A–C** and ESAM) constitute the additional transmembrane proteins. Zonula occludens (ZO) 1 and 2 are expressed cytoplasmic which can form a structural link to the actin cytoskeleton and associate with actin binding proteins.

The neurovascular unit (NVU) is comprised of numerous interacting cells that facilitate the formation, maintenance and functionality of both the BBB/iBRB. The presence of each cell type; astrocytes, pericytes, microglia and Müller cells, with innervation from neurones, are all required to maintain brain and retinal homeostasis. The endothelial cells that form the blood vessel lumen are surrounded by pericytes which are then ensheathed by astrocytic end-feet that forms a continuous layer with the basal lamina (Figure 1).

Astrocytes and Müller cells are the most common glial cells in the brain and retina, respectively. Both cells types have essential roles in maintaining tissue homeostasis (Abbott et al., 2006; Reichenbach and Bringmann, 2020). They are involved in (1) regulating ion and water transport due to influencing the expression and locality of influx and efflux transporters such as aquaporin-4, (2) microvascular permeability mediated by calcium signaling to the endothelium, (3) cell-cell communication via junctional components, (4) development and maintenance of the BBB/iBRB as loss of astrocytic end-feet overage leads to an increased BBB permeability (Segarra et al., 2018), and (5) release and uptake of neurotrophic factors such as glutamate and vascular endothelial growth factor (VEGF). Dysfunction of astrocyte or Müller cell behavior can contribute to neuroinflammation due to pro-inflammatory cytokine release or tissue edema due to water retention leading to tissue swelling (Abbott et al., 2006).

The presence of pericytes in the NVU aids in microvessel stability and regulation of blood flow due to pericyte contractility and relaxation (Peppiatt et al., 2006; Hamilton et al., 2010; Lendahl et al., 2019). In contrast to other vascular tissue beds, the ratio of pericytes to endothelial cells with the CNS and retina is significantly higher, with 1 pericyte:1 endothelial cell in the retina (Frank et al., 1990). Pericyte populations vary along the vasculature- differing in their morphology and alpha smooth muscle actin expression depending on their location. Pericytes and the endothelium are usually separated physically by the basement membrane although the two cell types can directly interact at peg-socket contact sites (Lendahl et al., 2019). Platelet-derived growth factor (PDGF) signaling recruits pericytes to the BBB and iBRB and if signaling becomes dysfunctional pericyte numbers are greatly reduced leading to increased barrier permeability and dysfunction, although this may become dispensable in adult mice (Armulik et al., 2010; Daneman et al., 2010b; Park et al., 2017). Pericytes also release factors, such as angiopoietin, that influence barrier properties by inducing tight junction protein expression (Hori et al., 2004). Loss or dysfunction of pericytes has been linked to various neurodegenerative conditions including, Alzheimer’s Disease, Amyotrophic lateral sclerosis (Lendahl et al., 2019) and Diabetic Retinopathy (Enge et al., 2002).

Monocyte-derived microglia are CNS-resident macrophages which become activated in response to any changes detected within their microenvironment, such as injury or inflammation. They continually undertake immune surveillance in the tissue they reside accounting for between 10 and 15% of the cell population (Perry et al., 2010; Ronaldson and Davis, 2020). Depending on the signaling pathway initiated microglia can become either pro-inflammatory (M1) or anti-inflammatory (M2) which can influence barrier properties by either upregulation or downregulation of tight junction components in both the brain and retina. M1 microglia have been implicated in BBB dysfunction due to the release and secretion of cytokines and chemokines such as interleukin (IL)- 1β, IL-12 and tumor necrosis factor (TNF)α, and CCL2 which can increase leukocyte extravasation. M2 microglia are believed to play a more protective role by controlling inflammation and resolving injury due to the release of cytokines, including IL-10 and transforming growth factor (TGF)-β (Ronaldson and Davis, 2020).

In addition to microglia, the presence of perivascular macrophages aids in maintaining tissue health. Perivascular macrophages act as antigen-presenting cells phagocytosing potential harmful material to present to leukocytes and subsequently can regulate leukocyte transmigration due to releasing anti-inflammatory cytokines. The presence of perivascular macrophages at the BBB and iBRB can enhance barrier tightness (Lapenna et al., 2018). As observed with the other cell types found within the NVU changes in perivascular macrophage behavior and number can be a causative role in neurodegenerative disease pathogenesis.

Basement membrane proteins are essential in supporting role for the cells found within the NVU which are derived from astrocytes, pericytes and the endothelium. In the brain there are two basement membranes- the endothelial and parenchymal basement membrane which under healthy conditions are indistinguishable from one another, keeping a separation between the endothelium and neurones/glial cells. Laminin, collagen IV, nidogen and heparin sulfate proteoglycans (HSPGs) are proteins that form the basement membrane and other additional proteins, such as fibronectin, are also present although their expression is dependent on the developmental or physiologically state (Thomsen et al., 2017). Agrin and perlecan are the most abundant HSPGs which integrate within the collagen IV and laminin network assisting in integrity of the basement membrane as well as having the capability to bind growth factors. The interaction of the basement membrane and the NVU cells is mediated by integrin or dystroglycan receptors that maintain the cells in their correct location. For example, collagen IV of the basement membrane interacts with endothelial β1 integrins. The expression of proteins found within the basement membrane network varies along the vascular beds. Laminin 411 and 511 are expressed in the endothelial basement membrane with low or patchy post-capillary venule expression of laminin 511 being preferential sites for leukocyte transmigration (Wu et al., 2009; Hallmann et al., 2020). Studies investigating neurodegenerative conditions, in conjunction with the use of transgenic animal models, have shown the important role that basement membrane proteins have in a functional and intact BBB (Thomsen et al., 2017). Many transgenic mice that lack the expression of a key basement membrane component are embryonic lethal, such as agrin or perlecan KO (Sarrazin et al., 2011) and collagen IV (Poschl et al., 2004), or die within a few weeks of birth, such as *Lama2−/−* mice (Miyagoe et al., 1997). Altered tight junction expression, resulting in a compromised BBB, can arise due to the loss of basement membrane components as seen in mice lacking astrocytic laminin (Yao et al., 2014).

## Tight Junctions

Tight junctions have been described to have “gate” (paracellular permeability) and “fence” (apical/basolateral polarity barrier) functions which are key to maintaining low endothelial permeability whilst providing a high transendothelial electrical resistance (Otani and Furuse, 2020). Individual cells can regulate the “tightness” of the junction depending on the cells physiological and pathological demands (Tsukita et al., 2001). The tight junction complex is formed from numerous interacting proteins and include the tight-junction-associated MARVEL proteins, claudin family members and junctional adhesion molecules (JAMs). These link to the actin cytoskeleton by a cytoplasmic plaque consisting of adaptor, scaffold and signaling proteins (Zihni et al., 2016). Tight junction complexes not only confer structural integrity but also play a role in numerous signaling pathways influencing their assembly, function and polarity as well as a role in gene expression (Zihni et al., 2016).

## Claudin Protein Family

The claudin protein family are integral transcellular components of tight junctions and considered to be the main structural components of intramembrane strands (Furuse et al., 1998; Tsukita et al., 2001). Claudins are a family of 27 proteins which form the primary junctional seal through homophilic or heterophilic interactions (Mineta et al., 2011). Claudins have numerous functions helping to establish barrier properties, restricting permeability to solutes and forming charge specific pores which permit ion diffusion (Zihni et al., 2016). It is believed that the functionality of claudin proteins is specified by the extracellular loop; the tightness and ion selectivity involves the first loop whilst the second loop is important for the two opposing membranes to interact and adhere (Krause et al., 2008). Ion selectivity of each molecule across the barrier is thought to be regulated by a specific claudin protein.

Claudin expression is tissue-specific, with many cells expressing more than one family member which can be altered in response to developmental stage. Junctional “tightness” and ion selectivity arises in response to the combination and ratio of claudin members (Liebner et al., 2000; Tsukita et al., 2001). Expression of Claudins-1, -3, -5, and -12 have been reported in the brain and retinal microvasculature. However, for both vascular beds claudin-5 appears to be the most highly enriched and may indeed be the only claudin expressed at high levels (Daneman et al., 2010a; Luo et al., 2011; Vanlandewijck et al., 2018).

## Claudin-5

Claudin-5 is expressed specifically on endothelial cells (Morita et al., 1999b), although during embryonic development it has been shown to be transiently expressed in the retinal pigment epithelium (Kojima et al., 2002). Due to its high enrichment at the BBB, the importance of claudin-5 in maintaining BBB function and integrity has been shown as claudin-5 null mice show a size-selective increase (for small molecules up to 800 Da) in BBB permeability and are embryonic lethal, dying within a few hours of birth (Nitta et al., 2003).

Alterations in claudin-5 expression have been implicated in a number of neurological conditions including schizophrenia, depression, epilepsy and traumatic brain injury (Doherty et al., 2016; Menard et al., 2017; Greene et al., 2018, 2020; Farrell et al., 2019). In addition, claudin-5 remodeling occurs at sites of leukocyte transmigration in both physiological and pathological conditions such as Multiple Sclerosis (Paul et al., 2013; Winger et al., 2014; Castro Dias et al., 2021) and mislocalization of claudin-5 occurs in a mouse model of oxygen induced retinopathy (Luo et al., 2011). Recent work has found the inner retinal blood vessels to be highly dynamic with claudin-5 expression regulated in a circadian-manner and claudin-5 changes being a key mediator in initiating dry age-related macular degeneration like pathology (Hudson et al., 2019). Transient modulation of claudin-5 expression using RNA interference has been shown to be beneficial in animal models of traumatic brain injury, Alzheimer’s disease and choroidal neovascularization (Campbell et al., 2009, 2012; Keaney et al., 2015). This technique enabled either the removal of neurotoxic material from brain to blood or the enhanced penetration and efficacy of small molecule therapeutics into the brain or retina. Claudin-5 expression can be modulated by a number of factors including glucocorticoids, hypoxia, hormones and VEGF-A (Koto et al., 2007; Argaw et al., 2009; Burek et al., 2010; Hudson et al., 2014).

## Claudin-1

Claudin-1 is ubiquitously expressed in most tissues of the body (Furuse et al., 1998), with a key role in skin barrier formation found as claudin-1 knockout mice die of dehydration due to the loss of the junctional barrier function to water and macromolecules (Furuse et al., 2002). The requirement of claudin-1 in tight junctions of the BBB is highly debated and may vary among different species. In response to pathological conditions claudin-1 expression can be altered leading to BBB disruption. Claudin-1 upregulation has been found in conditions such as stroke (Sladojevic et al., 2019), where it appears to impair interactions with other tight junction components due to its incorporation. In human glioblastoma multiforme claudin-1 was found to be downregulated in tumor vessels (Liebner et al., 2000). In contrast, several groups have shown that claudin-1 mRNA is not detected in brain endothelial cells (Pfeiffer et al., 2011; Vanlandewijck et al., 2018). It was found that in an animal model of multiple sclerosis, experimental autoimmune encephalomyelitis (EAE), endothelial specific inducible ectopic BBB expression of claudin-1 reduced BBB permeability and ameliorated clinical disease signs (Pfeiffer et al., 2011).

## Claudin-3

The role of claudin-3 in BBB integrity was first shown in studies investigating EAE and glioblastoma multiforme where loss of expression lead to a loss of BBB function (Wolburg et al., 2003). Maturation and stabilization of barrier properties occurred in response to β-catenin induced claudin-3 expression (Liebner et al., 2008). However recent work utilizing claudin-3 deficient mice and transcriptomic analysis found claudin-3 was not expressed in the BBB endothelium (Vanlandewijck et al., 2018; Castro Dias et al., 2019b). It has been suggested that the detection of claudin-3 at the BBB may arise due to issues with antibody specificity and cross-reactivity.

## Claudin-12

Claudin-12 is an atypical claudin family member which is unable to interact with the cytoskeleton due to the inability to bind to accessory adaptor proteins as it lacks a PDZ binding motif. Claudin-12 is expressed in numerous organs, and has been described to be present in the BBB (Nitta et al., 2003) and in the retina (Luo et al., 2011), although its role in the BBB tight junction complex was not fully elucidated. Recent work has found that brain claudin-12 expression is predominantly found in neurons, astrocytes and smooth muscle cells rather than the endothelium (Vanlandewijck et al., 2018; Castro Dias et al., 2019a). In addition, loss of claudin-12 did not impact BBB integrity under physiological or pathological inflammatory conditions such as EAE. Mice lacking claudin-12 do show some behavioral deficits including decreased locomotion and decreased anxiety, along with minor ear and retina phenotypes such as slight changes in hearing sensitivity and a reduction in axial length in the eye (Castro Dias et al., 2019a).

## Other Claudin Family Members

Additional claudin family members have been suggested to be expressed at the BBB, although their cellular expression and importance in barrier integrity has not been fully characterized. This is also true for claudin expression in the retina with some family members being expressed in a developmental manner (Luo et al., 2011). Claudin-4 is integral in maintaining astrocytic tight junctions and claudin-4 degradation influences EAE development (Horng et al., 2017). Recent studies has suggested claudin-4 to be a novel BBB tight junction component (Berndt et al., 2019), however, single cell RNA sequencing data could not detect claudin-4 expression in any brain cell types (He et al., 2018; Vanlandewijck et al., 2018). Expression of claudin-11 has been detected in a co-culture primary BBB model of endothelial cells, glial cell and pericytes (Bocsik et al., 2016) as well as in microdissected mouse and human cortical capillaries (Berndt et al., 2019). However, claudin-11 expression may appear to be more specific for oligodendrocytes localizing within the myelin rather than the tight junctions (Bronstein et al., 1996; Morita et al., 1999a; Vanlandewijck et al., 2018). Claudin-20 and -25 have also been implicated as BBB tight junction components (Berndt et al., 2019) although single cell RNA sequencing data detected claudin-20 at very low levels within capillary and arterial endothelial cells and astrocytes and claudin-25 expressed highest in oligodendrocytes (Vanlandewijck et al., 2018).

## Tight-Junction-Associated Marvel Proteins

The tight-junction-associated marvel proteins (TAMP) family of proteins include occludin, tricellulin (MARVEL D2) and MARVEL D3. Occludin was the first integral membrane protein identified to localize to tight junctions (Furuse et al., 1993) and its high expression at the BBB endothelium correlates with low endothelial permeability (Hirase et al., 1997). In contrast to claudin-5 null mice, mice lacking occludin are viable and do not have a deficient BBB due to the presence of morphologically intact tight junctions (Saitou et al., 2000; Tsukita et al., 2001). This suggests that occludin may play more of a regulatory, rather than structural, role in paracellular permeability, which can be compensated for by other tight junction proteins. The phosphorylation status of occludin is important for barriergenesis aiding in formation (Sakakibara et al., 1997), permeability (Antonetti et al., 1999; Harhaj et al., 2006) and tight junction trafficking (Murakami et al., 2009). Occludin domains exhibit distinct functions and regulatory features (Cummins, 2012). The C-terminus of occludin associates with the actin cytoskeleton via accessory proteins, such as zonula occluden (ZO)-1 (Furuse et al., 1994) and is important for paracellular permeability along with essential signaling properties. The phosphorylation status of occludin is important in disease pathology- in response to diabetes an increase in VEGF mediated phosphorylation of occludin leads to a loss of iBRB integrity and subsequent vision loss (Antonetti et al., 1998; Goncalves et al., 2021).

Tricellulin (MARVEL D2) is another transmembrane protein that is normally localized to tricellular junctions in the brain and retina (Ikenouchi et al., 2005; Iwamoto et al., 2014). However, tricellulin relocates to bicellular junctions in the absence of occludin (Ikenouchi et al., 2008). Therefore, tricellulin may have a compensatory role in the absence of occludin in the bicellular tight junction formation. Several studies have found tricellulin to be specifically enriched in brain endothelial cells (Daneman et al., 2010a; Vanlandewijck et al., 2018; Castro Dias et al., 2021). Similar to mice lacking occludin, tricellulin-deficient mice are viable although they develop hearing loss (Kitajiri et al., 2014; Kamitani et al., 2015). Under inflammatory conditions within the brain endothelium tricellulin expression is reduced leading to increased leukocyte transmigration in response to destabilization of both bi- and tricellular junctions (Castro Dias et al., 2021).

MARVEL D3 is a transmembrane protein which lacks the C-terminus found in occludin and tricellulin (Steed et al., 2009; Raleigh et al., 2010). The role of MARVEL D3 at the BBB and iBRB is still unknown although it has been found to be down-regulated in response to oxygen-glucose deprivation (Tornabene et al., 2019).

## Lipolysis-Stimulated Lipoprotein Receptor (LSR/angulin-1)

LSR recruits tricellulin to tricellular tight junctions (Masuda et al., 2011) and has been found to be specifically expressed in the BBB and iBRB (Daneman et al., 2010a; Iwamoto et al., 2014; Sohet et al., 2015). Mice deficient for *LSR* are embryonic lethal (Mesli et al., 2004) and show impaired barriergenesis as the BBB fails to seal and is leaky to small molecules (Sohet et al., 2015). As found for tricellulin, expression of LSR was found to be down-regulated in response to inflammation, such as EAE, and middle cerebral artery occlusion which led to destabilization of the bi- and tri-cellular junctions (Sohet et al., 2015; Castro Dias et al., 2021).

## Zonula Occludens

Zonula occludens (ZO) proteins are cytoplasmic plaque proteins that form a structural link to the actin cytoskeleton and can bind to actin binding proteins including α-catenin and cortactin (Pachter et al., 2003). ZO-1 was the first tight junction protein to be discovered in both epithelial and endothelial cells, although only the ZO-1 α^–^ form is expressed in endothelial cells (Stevenson et al., 1986; Balda and Anderson, 1993). ZO-2 and ZO-3, have similar sequence homology to ZO-1, also localizing to tight junctions (Zihni et al., 2016), although ZO-3 is not expressed in BBB tight junctions (Inoko et al., 2003). Cells deficient for ZO-1/2 fail to form tight junctions showing the importance of ZO proteins for tight junction assembly (Umeda et al., 2006) while ZO-1 knockout mice are embryonic lethal which is believed to be due to ZO-1 importance in endothelial tissue organization (Katsuno et al., 2008). ZO proteins have specific domains that allow for various protein-protein interactions; PDZ domains enable ZO-1 to interact with ZO-2, ZO-3, and claudin family C-terminus and occludin interacts via guanylate cyclase domain (Itoh et al., 1999).

In addition to tight junction complex formation, ZO-1 and ZO-2 have a role in gene transcription regulating transcription factors as well as cell proliferation via its ability to bind ZO-1-associated nucleic acid binding (ZONAB) (Balda and Matter, 2009). Accumulation of ZONAB in the nucleus occurs when cell density is low, but if cell density is high ZONAB interacts with ZO-1 at cellular junctions (Balda and Matter, 2000; Balda et al., 2003). ZO-1 has also been found to mediate a role in endothelial cell-cell tension, cell migration and angiogenesis (Tornavaca et al., 2015). Like claudin-5 and occludin, ZO-1 expression is reduced in certain neurological diseases leading to barrier instability.

## Junctional Adhesion Molecules

JAMs are single span members of the immunoglobulin superfamily (Martìn-Padura et al., 1998) that are important for tight junction assembly and integrity (Ebnet, 2017). There are three family members JAM-A, -B, and -C which can all interact with PAR-3, a core component of the cellular polarity regulating machinery which localizes to tight junctions (Ebnet, 2017). All three JAMs have the capacity to interact with ZO-1, while JAM-A can also regulate the localization of ZO-1 within the junction complex. JAM-A is the predominant isoform in the brain and retinal endothelium regulating permeability changes (Aurrand-Lions et al., 2001; Tomi and Hosoya, 2004). Furthermore, JAM-A and JAM-C have been implicated in leukocyte trafficking as well as junction integrity (Woodfin et al., 2007, 2011; Williams et al., 2015; Hou et al., 2021). JAM-C has been shown to play a specific role in regulating microvascular permeability during inflammation by targeting the adherens junction protein vascular endothelial cadherin which can regulate claudin-5 expression via a β-catenin and FoxO1 dependent pathway (Taddei et al., 2008).

Endothelial selective cell adhesion molecule (ESAM) has a similar structure to JAM proteins. ESAM localization is supported by its interaction with ZO-1 in the brain capillaries (Nasdala et al., 2002) and it plays a role in endothelial cell-cell interaction during vascular development and neutrophil extravasation during early stages of inflammation (Wegmann et al., 2004).

## Conclusion

Tight junctions found in the BBB and iBRB are complex and dynamic in nature, comprising numerous interacting proteins that aid in the gate and fence function. The contribution of other cell types found in the NVU, astrocytes, pericytes, and microglia/macrophages as well as the presence of the basement membrane are essential in ensuring the highly specialized barrier properties. All components are integral in maintaining a homeostatic balance and the integrity of the brain and retina in both healthy and disease states. Of particular importance in maintaining BBB and iBRB integrity is claudin-5, the most highly enriched tight junction component which when dysregulated has been linked to a number of neurodegenerative pathologies. In recent years the involvement of claudin–1, –3, and –12 in BBB integrity and function has come into dispute as these claudin family members are found to be expressed at extremely low levels in the brain endothelium.

## Author Contributions

NH and MC wrote the manuscript. Both authors contributed to the article and approved the submitted version.

## Conflict of Interest

The authors declare that the research was conducted in the absence of any commercial or financial relationships that could be construed as a potential conflict of interest.

## Publisher’s Note

All claims expressed in this article are solely those of the authors and do not necessarily represent those of their affiliated organizations, or those of the publisher, the editors and the reviewers. Any product that may be evaluated in this article, or claim that may be made by its manufacturer, is not guaranteed or endorsed by the publisher.

## References

[B1] Abbott N. J. Ronnback L. Hansson E. (2006). Astrocyte-endothelial interactions at the blood-brain barrier. *Nat. Rev. Neurosci.* 7 41–53. 10.1038/nrn1824 16371949

[B2] Antonetti D. A. Barber A. J. Hollinger L. A. Wolpert E. B. Gardner T. W. (1999). Vascular endothelial growth factor induces rapid phosphorylation of tight junction proteins occludin and zonula occluden 1. A potential mechanism for vascular permeability in diabetic retinopathy and tumors. *J. Biol. Chem.* 274 23463–23467. 10.1074/jbc.274.33.23463 10438525

[B3] Antonetti D. A. Barber A. J. Khin S. Lieth E. Tarball J. M. Gardner T. W. (1998). Vascular permeability in experimental diabetes is associated with reduced endothelial occludin content: vascular endothelial growth factor decreases occludin in retinal endothelial cells. Penn State Retina Research Group. *Diabetes* 47 1953–1959. 10.2337/diabetes.47.12.1953 9836530

[B4] Argaw A. T. Gurfein B. T. Zhang Y. Zameer A. John G. R. (2009). VEGF-mediated disruption of endothelial CLN-5 promotes blood-brain barrier breakdown. *Proc. Natl. Acad. Sci. U. S. A.* 106 1977–1982. 10.1073/pnas.0808698106 19174516 PMC2644149

[B5] Armulik A. Genové G. Mäe M. Nisancioglu M. H. Wallgard E. Niaudet C. (2010). Pericytes regulate the blood brain barrier. *Nature* 468 557–561. 20944627 10.1038/nature09522

[B6] Aurrand-Lions M. Duncan L. Ballestrem C. Imhof B. A. (2001). JAM-2, a novel immunoglobulin superfamily molecule, expressed by endothelial and lymphatic cells. *J. Biol. Chem.* 276 2733–2741. 10.1074/jbc.m005458200 11053409

[B7] Balda M. S. Anderson J. M. (1993). Two classes of tight junctions are revealed by ZO-1 isoforms. *Am. J. Physiol.* 264 C918–C924. 10.1152/ajpcell.1993.264.4.C918 7682777

[B8] Balda M. S. Garrett M. D. Matter K. (2003). The ZO-1-associated Y-box factor ZONAB regulates epithelial cell proliferation and cell density. *J. Cell Biol.* 160 423–432. 10.1083/jcb.200210020 12566432 PMC2172662

[B9] Balda M. S. Matter K. (2000). The tight junction protein ZO-1 and an interacting transcription factor regulate ErbB-2 expression. *EMBO J.* 19 2024–2033. 10.1093/emboj/19.9.2024 10790369 PMC305688

[B10] Balda M. S. Matter K. (2009). Tight junctions and the regulation of gene expression. *Biochim. Biophys. Acta* 1788 761–767. 10.1016/j.bbamem.2008.11.024 19121284

[B11] Berndt P. Winkler L. Cording J. Breitkreuz-Korff O. Rex A. Dithmer S. (2019). Tight junction proteins at the blood–brain barrier: far more than claudin-5. *Cell. Mol. Life Sci.* 76 1987–2002. 10.1007/s00018-019-03030-7 30734065 PMC11105330

[B12] Bocsik A. Walter F. R. Gyebrovszki A. Fülöp L. Blasig I. Dabrowski S. (2016). Reversible Opening of Intercellular Junctions of Intestinal Epithelial and Brain Endothelial Cells With Tight Junction Modulator Peptides. *J. Pharm. Sci*. 105 754–765. 10.1016/j.xphs.2015.11.018 26869428

[B13] Bronstein J. M. Popper P. Micevych P. E. Farber D. B. (1996). Isolation and characterization of a novel oligodendrocyte-specific protein. *Neurology* 47 772–778. 10.1212/wnl.47.3.772 8797478

[B14] Burek M. Arias-Loza P. A. Roewer N. Förster C. Y. (2010). Claudin-5 as a novel estrogen target in vascular endothelium. *Arterioscler. Thromb. Vasc. Biol.* 30 298–304. 10.1161/ATVBAHA.109.197582 19910637

[B15] Campbell M. Hanrahan F. Gobbo O. L. Kelly M. E. Kiang A. S. Humphries M. M. (2012). Targeted suppression of claudin-5 decreases cerebral oedema and improves cognitive outcome following traumatic brain injury. *Nat. Commun*. 3:849. 10.1038/ncomms1852 22617289

[B16] Campbell M. Nguyen A. T. Kiang A. S. Tam L. C. S. Gobbo O. L. Kerskens C. (2009). An experimental platform for systemic drug delivery to the retina. *Proc. Natl. Acad. Sci. U. S. A*. 106 17817–17822. 10.1073/pnas.0908561106 19822744 PMC2760489

[B17] Castro Dias M Quesada A. O. Soldati S. Bösch F. Gruber I. Hildbrand T. (2021). Brain endothelial tricellular junctions as novel sites for T cell diapedesis across the blood–brain barrier. *J. Cell Sci.* 134:jcs253880. 10.1242/jcs.253880 33912914 PMC8121105

[B18] Castro Dias M. Coisne C. Lazarevic I. Baden P. Hata M. Iwamoto N. (2019b). Claudin-3-deficient C57BL/6J mice display intact brain barriers. *Sci. Rep*. 9:203. 10.1038/s41598-018-36731-3 PMC6338742 30659216

[B19] Castro Dias M. Coisne C. Baden P. Enzmann G. Garrett L. Becker L. (2019a). Claudin-12 is not required for blood-brain barrier tight junction function. *Fluids Barriers CNS* 16:30. 10.1186/s12987-019-0150-9 PMC6739961 31511021

[B20] Cummins P. M. (2012). Occludin: one protein, many forms. *Mol. Cell Biol*. 32 242–250. 22083955 10.1128/MCB.06029-11 PMC3255790

[B21] Daneman R. Zhou L. Kebede A. A. Barres B. A. (2010b). Pericytes are required for blood-brain barrier integrity during embryogenesis. *Nature* 468 562–566. 10.1038/nature09513 20944625 PMC3241506

[B22] Daneman R. Zhou L. Agalliu D. Cahoy J. D. Kaushal A. Barres B. A. (2010a). The mouse blood-brain barrier transcriptome: a new resource for understanding the development and function of brain endothelial cells. *PLoS One* 5:e13741. 10.1371/journal.pone.0013741 21060791 PMC2966423

[B23] Doherty C. P. O’Keefe E. Wallace E. Loftus T. Keaney J. Kealy J. (2016). Blood-Brain Barrier Dysfunction as a Hallmark Pathology in Chronic Traumatic Encephalopathy. *J. Neuropathol. Exp. Neurol*. 75 656–662. 27245243 10.1093/jnen/nlw036 PMC4913433

[B24] Ebnet K. (2017). Junctional Adhesion Molecules (JAMs): cell Adhesion Receptors With Pleiotropic Functions in Cell Physiology and Development. *Physiol. Rev.* 97 1529–1554. 10.1152/physrev.00004.2017 28931565

[B25] Enge M. Bjarnegard M. Gerhardt H. Gustafsson E. Kalen M. Asker N. (2002). Endothelium-specific platelet-derived growth factor-B ablation mimics diabetic retinopathy. *EMBO J.* 21 4307–4316. 10.1093/emboj/cdf418 12169633 PMC126162

[B26] Farrell M. Aherne S. O’Riordan S. O’Keeffe E. Greene C. Campbell M. (2019). Blood-brain barrier dysfunction in a boxer with chronic traumatic encephalopathy and schizophrenia. *Clin. Neuropathol.* 38 51–58. 10.5414/NP301130 30574863

[B27] Frank R. N. Turczyn T. J. Das A. (1990). Pericyte coverage of retinal and cerebral capillaries. *Invest. Ophthalmol. Vis. Sci.* 31 999–1007. 2354923

[B28] Furuse M. Fujita K. Hiiragi T. Fujimoto K. Tsukita S. (1998). Claudin-1 and -2: novel integral membrane proteins localizing at tight junctions with no sequence similarity to occludin. *J. Cell Biol*. 141 1539–1550. 10.1083/jcb.141.7.1539 9647647 PMC2132999

[B29] Furuse M. Hata M. Furuse K. Yoshida Y. Haratake A. Sugitani Y. (2002). Claudin-based tight junctions are crucial for the mammalian epidermal barrier: a lesson from claudin-1-deficient mice. *J. Cell Biol.* 156 1099–1111. 10.1083/jcb.200110122 11889141 PMC2173463

[B30] Furuse M. Hirase T. Itoh M. Nagafuchi A. Yonemura S. Tsukita S. (1993). Occludin: a novel integral membrane protein localizing at tight junctions. *J. Cell Biol.* 123 1777–1788. 10.1083/jcb.123.6.1777 8276896 PMC2290891

[B31] Furuse M. Itoh M. Hirase T. Nagafuchi A. Yonemura S. Tsukita S. (1994). Direct association of occludin with ZO-1 and its possible involvement in the localization of occludin at tight junctions. *J. Cell Biol.* 127 1617–1626. 10.1083/jcb.127.6.1617 7798316 PMC2120300

[B32] Goncalves A. Dreffs A. Lin C. M. Sheskey S. Hudson N. Keil J. (2021). Vascular Expression of Permeability-Resistant Occludin Mutant Preserves Visual Function in Diabetes. *Diabetes* 70 1549–1560. 10.2337/db20-1220 33883214 PMC8336002

[B33] Greene C. Hanley N. Campbell M. (2020). Blood-brain barrier associated tight junction disruption is a hallmark feature of major psychiatric disorders. *Transl. Psychiatry* 10:373. 10.1038/s41398-020-01054-3 33139732 PMC7606459

[B34] Greene C. Kealy J. Humphries M. M. Gong Y. Hou J. Hudson N. (2018). Dose-dependent expression of claudin-5 is a modifying factor in schizophrenia. *Mol. Psychiatry* 23 2156–2166. 10.1038/mp.2017.156 28993710 PMC6298981

[B35] Hallmann R. Hannocks M. J. Song J. Zhang X. Di Russo J. Luik A. L. (2020). The role of the basement membrane laminins in vascular function. *Int. J. Biochem. Cell Biol.* 127:105823. 10.1016/j.biocel.2020.105823 32781135

[B36] Hamilton N. B. Atwell D. Hall C. N. (2010). Pericyte-mediated regulation of capillary diameter: a component of neurovascular coupling in health and disease. *Front. Neuroenergetics* 2:5. 10.3389/fnene.2010.00005 20725515 PMC2912025

[B37] Harhaj N. S. Felinski E. A. Wolpert E. B. Sundstrom J. M. Gardner T. W. Antonetti D. (2006). VEGF activation of protein kinase C stimulates occludin phosphorylation and contributes to endothelial permeability. *Invest. Ophthalmol. Vis. Sci.* 47 5106–5115. 17065532 10.1167/iovs.06-0322

[B38] He L. Vanlandewijck M. Mäe M. A. Andrae J. Ando K. Del Gaudio F. (2018). Single-cell RNA sequencing of mouse brain and lung vascular and vessel-associated cell types. *Sci. Data* 5:180160. 10.1038/sdata.2018.160 30129931 PMC6103262

[B39] Hirase T. Staddon J. M. Saitou M. Ando-Akatsuka Y. Itoh M. Furuse M. (1997). Occludin as a possible determinant of tight junction permeability in endothelial cells. *J. Cell Sci*. 110 1603–1613. 10.1242/jcs.110.14.1603 9247194

[B40] Hori S. Ohtsuki S. Hosoya K. Nakashima E. Terasaki T. (2004). A pericyte-derived angiopoietin-1 multimeric complex induces occludin gene expression in brain capillary endothelial cells through Tie-2 activation in vitro. *J. Neurochem*. 89 503–513. 10.1111/j.1471-4159.2004.02343.x 15056293

[B41] Horng S. Therattil A. Moyon S. Gordon A. Kim K. Argaw A. T. (2017). Astrocytic tight junctions control inflammatory CNS lesion pathogenesis. *J. Clin. Invest*. 127 3136–3151. 10.1172/JCI91301 28737509 PMC5531407

[B42] Hou X. Du H. J. Zhou J. Hu D. Wang Y. S. Li X. (2021). Role of Junctional Adhesion Molecule C in the Regulation of Inner Endothelial Blood-Retinal Barrier Function. *Front. Cell Dev. Biol.* 9:695657. 10.3389/fcell.2021.695657 34164405 PMC8215391

[B43] Hudson N. Campbell M. (2019). Inner Blood-Retinal Barrier Regulation in Retinopathies. *Adv. Exp. Med. Biol*. 1185 329–333. 10.1007/978-3-030-27378-1_54 31884633

[B44] Hudson N. Celkova L. Hopkins A. Greene C. Storti F. Ozaki E. (2019). Dysregulated claudin-5 cycling in the inner retina causes retinal pigment epithelial cell atrophy. *JCI Insight* 4:e130273. 10.1172/jci.insight.130273 31391341 PMC6693834

[B45] Hudson N. Powner M. B. Sarker M. H. Burgoyne T. Campbell M. Ockrim Z. K. (2014). Differential apicobasal VEGF signaling at vascular blood-neural barriers. *Dev. Cell* 30 541–552. 10.1016/j.devcel.2014.06.027 25175707 PMC4160345

[B46] Ikenouchi J. Furuse M. Furuse K. Sasaki H. Tsukita S. Tsukita S. (2005). Tricellulin constitutes a novel barrier at tricellular contacts of epithelial cells. *J. Cell Biol*. 171 939–945. 10.1083/jcb.200510043 16365161 PMC2171318

[B47] Ikenouchi J. Sasaki H. Tsukita S. Furuse M. Tsukita S. (2008). Loss of occludin affects tricellular localization of tricellulin. *Mol. Biol. Cell* 19 4687–4693. 10.1091/mbc.e08-05-0530 18768749 PMC2575184

[B48] Inoko A. Itoh M. Tamura A. Matsuda M. Furuse M. Tsukita S. (2003). Expression and distribution of ZO-3, a tight junction MAGUK protein, in mouse tissues. *Genes Cells* 8 837–845. 10.1046/j.1365-2443.2003.00681.x 14622136

[B49] Itoh M. Furuse M. Morita K. Kubota K. Saitou M. Tsukita S. (1999). Direct binding of three tight junction-associated MAGUKs, ZO-1, ZO-2, and ZO-3, with the COOH termini of claudins. *J. Cell Biol*. 147 1351–1363. 10.1083/jcb.147.6.1351 10601346 PMC2168087

[B50] Iwamoto N. Higashi T. Furuse M. (2014). Localization of angulin-1/LSR and tricellulin at tricellular contacts of brain and retinal endothelial cells in vivo. *Cell Struct. Funct.* 39 1–8. 10.1247/csf.13015 24212375

[B51] Kamitani T. Sakaguchi H. Tamura A. Miyashita T. Yamazaki Y. Tokumasu R. (2015). Deletion of Tricellulin Causes Progressive Hearing Loss Associated with Degeneration of Cochlear Hair Cells. *Sci. Rep*. 5:18402. 10.1038/srep18402 26677943 PMC4683410

[B52] Katsuno T. Umeda K. Matsui T. Hata M. Tamura A. Itoh M. (2008). Deficiency of zonula occludens-1 causes embryonic lethal phenotype associated with defected yolk sac angiogenesis and apoptosis of embryonic cells. *Mol. Biol. Cell* 19 2465–2475. 10.1091/mbc.e07-12-1215 18353970 PMC2397322

[B53] Keaney J. Walsh D. M. O’Malley T. Hudson N. Crosbie D. E. Loftus T. (2015). Autoregulated paracellular clearance of amyloid-β across the blood-brain barrier. *Sci. Adv*. 1:e1500472. 10.1126/sciadv.1500472 26491725 PMC4610013

[B54] Kitajiri S. Katsuno T. Sasaki H. Ito J. Furuse M. Tsukita S. (2014). Deafness in occludin-deficient mice with dislocation of tricellulin and progressive apoptosis of the hair cells. *Biol. Open* 3 759–766. 10.1242/bio.20147799 25063198 PMC4133728

[B55] Kojima S. Rahner C. Peng S. Rizzolo L. J. (2002). Claudin 5 is transiently expressed during the development of the retinal pigment epithelium. *J. Membr. Biol*. 186 81–88. 10.1007/s00232-001-0137-7 11944085

[B56] Koto T. Takubo K. Ishida S. Shinoda H. Inoue M. Tsubota K. (2007). Hypoxia disrupts the barrier function of neural blood vessels through changes in the expression of claudin-5 in endothelial cells. *Am. J. Pathol.* 170 1389–1397. 10.2353/ajpath.2007.060693 17392177 PMC1829471

[B57] Krause G. Winkler L. Mueller S. L. Haseloff R. F. Piontek J. Blasig I. E. (2008). Structure and function of claudins. *Biochim. Biophys. Acta* 1778 631–645. 10.1016/j.bbamem.2007.10.018 18036336

[B58] Lapenna A. De Palma M. Lewis C. E. (2018). Perivascular macrophages in health and disease. *Nat. Rev. Immunol.* 18 689–702. 10.1038/s41577-018-0056-9 30127389

[B59] Lendahl U. Nilsson P. Betsholtz C. (2019). Emerging links between cerebrovascular and neurodegenerative diseases- a special role for pericytes. *EMBO Rep*. 20:e48070. 10.15252/embr.201948070 31617312 PMC6831996

[B60] Liebner S. Corada M. Bangsow T. Babbage J. Taddei A. Czupalla C. J. (2008). Wnt/beta-catenin signaling controls development of the blood-brain barrier. *J. Cell Biol.* 183 409–417. 10.1083/jcb.200806024 18955553 PMC2575783

[B61] Liebner S. Fischmann A. Rascher G. Duffner F. Grote E. H. Kalbacher H. (2000). Claudin-1 and claudin-5 expression and tight junction morphology are altered in blood vessels of human glioblastoma multiforme. *Acta Neuropathol.* 100 323–331. 10.1007/s004010000180 10965803

[B62] Luo Y. Xiao W. Zhu X. Mao Y. Liu X. Huang J. (2011). Differential expression of claudins in retinas during normal development and the angiogenesis of oxygen-induced retinopathy. *Invest. Ophthalmol. Vis. Sci.* 52 7556–7564. 10.1167/iovs.11-7185 21862644

[B63] Martìn-Padura I. Lostaglio S. Schneemann M. Williams L. Romano M. Fruscella P. (1998). Junctional adhesion molecule, a novel member of the immunoglobulin superfamily that distributes at intercellular junctions and modulates monocyte transmigration. *J. Cell Biol*. 142 117–127. 10.1083/jcb.142.1.117 9660867 PMC2133024

[B64] Masuda S. Oda Y. Sasaki H. Ikenouchi J. Higashi T. Akashi M. (2011). LSR defines cell corners for tricellular tight junction formation in epithelial cells. *J. Cell Sci*. 124 548–555. 10.1242/jcs.072058 21245199

[B65] Menard C. Pfau M. L. Hodes G. E. Kana V. Wang V. X. Bouchard S. (2017). Social stress induces neurovascular pathology promoting depression. *Nat. Neurosci*. 20 1752–1760. 29184215 10.1038/s41593-017-0010-3 PMC5726568

[B66] Mesli S. Javorschi S. Bérard A. M. Landry M. Priddle H. Kivlichan D. (2004). Distribution of the lipolysis stimulated receptor in adult and embryonic murine tissues and lethality of LSR-/- embryos at 12.5 to 14.5 days of gestation. *Eur. J. Biochem*. 271 3103–3114. 10.1111/j.1432-1033.2004.04223.x 15265030

[B67] Mineta K. Yamamoto Y. Yamazaki Y. Tanaka H. Tada Y. Saitou K. (2011). Predicted expansion of the claudin multigene family. *FEBS Lett*. 585 606–612. 10.1016/j.febslet.2011.01.028 21276448

[B68] Miyagoe Y. Hanaoka K. Nonaka I. Hayasaka M. Nabeshima Y. Arahata K. (1997). Laminin alpha2 chain-null mutant mice by targeted disruption of the Lama2 gene: a new model of merosin (laminin 2)-deficient congenital muscular dystrophy. *FEBS Lett.* 415 33–39. 10.1016/s0014-5793(97)01007-7 9326364

[B69] Morita K. Sasaki H. Furuse M. Tsukita S. (1999b). Endothelial claudin: claudin-5/TMVCF constitutes tight junction strands in endothelial cells. *J. Cell Biol.* 147 185–194. 10.1083/jcb.147.1.185 10508865 PMC2164984

[B70] Morita K. Sasaki H. Fujimoto K. Furuse M. Tsukita S. (1999a). Claudin-11/OSP-based tight junctions of myelin sheaths in brain and Sertoli cells in testis. *J. Cell Biol*. 145 579–588. 10.1083/jcb.145.3.579 10225958 PMC2185072

[B71] Murakami T. Felinski E. A. Antonetti D. A. (2009). Occludin phosphorylation and ubiquitination regulate tight junction trafficking and vascular endothelial growth factor-induced permeability. *J. Biol. Chem.* 284 21036–21046. 10.1074/jbc.m109.016766 19478092 PMC2742868

[B72] Nasdala I. Wolburg-Buchholz K. Wolburg H. Kuhn A. Ebnet K. Brachtendorf G. (2002). A transmembrane tight junction protein selectively expressed on endothelial cells and platelets. *J. Biol. Chem.* 277 16294–16303. 11847224 10.1074/jbc.M111999200

[B73] Nitta T. Hata M. Gotoh S. Seo Y. Sasaki H. Hashimoto N. (2003). Size-selective loosening of the blood-brain barrier in claudin-5-deficient mice. *J. Cell Biol.* 161 653–660. 10.1083/jcb.200302070 12743111 PMC2172943

[B74] Otani T. Furuse M. (2020). Tight Junction Structure and Function Revisited. *Trends Cell Biol*. 30 805–817. 32891490 10.1016/j.tcb.2020.08.004

[B75] Pachter J. S. de Vries H. E. Fabry Z. (2003). The blood-brain barrier and its role in immune privilege in the central nervous system. *J. Neuropathol. Exp. Neurol*. 62 593–604. 12834104 10.1093/jnen/62.6.593

[B76] Park D. Y. Lee J. Kim J. Kim K. Hong S. Han S. (2017). Plastic roles of pericytes in the blood-retinal barrier. *Nat. Commun.* 8:15296. 10.1038/ncomms15296 28508859 PMC5440855

[B77] Paul D. Cowan A. E. Ge S. Pachter J. S. (2013). Novel 3D analysis of Claudin-5 reveals significant endothelial heterogeneity among CNS microvessels. *Microvasc. Res*. 86 1–10. 10.1016/j.mvr.2012.12.001 23261753 PMC3570614

[B78] Peppiatt C. M. Howarth C. Mobbs P. Atwell D. (2006). Bidirectional control of CNS capillary diameter by pericytes. *Nature* 443 700–704. 10.1038/nature05193 17036005 PMC1761848

[B79] Perry V. H. Nicoll J. A. Holmes C. (2010). Microglia in neurodegenerative disease. *Nat. Rev. Neurol.* 6 193–201. 20234358 10.1038/nrneurol.2010.17

[B80] Pfeiffer F. Schäfer J. Lyck R. Makrides V. Brunner S. Schaeren-Wiemers N. (2011). Claudin-1 induced sealing of blood-brain barrier tight junctions ameliorates chronic experimental autoimmune encephalomyelitis. *Acta Neuropathol.* 122 601–614. 10.1007/s00401-011-0883-2 21983942 PMC3207130

[B81] Poschl E. Schlotzer-Schrehardt U. Brachvogel B. Saito K. Ninomiya Y. Mayer U. (2004). Collagen IV is essential for basement membrane stability but dispensable for initiation of its assembly during early development. *Development* 131 1619–1628. 10.1242/dev.01037 14998921

[B82] Raleigh D. R. Marchiando A. M. Zhang Y. Shen L. Sasaki H. Wang Y. (2010). Tight Junction–associated MARVEL Proteins MarvelD3, Tricellulin, and Occludin Have Distinct but Overlapping Functions. *Mol. Biol. Cell* 21 1200–1213. 10.1091/mbc.e09-08-0734 20164257 PMC2847524

[B83] Reichenbach A. Bringmann A. (2020). Glia of the human retina. *Glia* 68 768–796. 31793693 10.1002/glia.23727

[B84] Ronaldson P. T. Davis T. P. (2020). Regulation of blood-brain barrier integrity by microglia in health and disease: a therapeutic opportunity. *J. Cereb. Blood Flow Metab.* 40 S6–S24. 10.1177/0271678X20951995 32928017 PMC7687032

[B85] Saitou M. Furuse M. Sasaki H. Schulzke J. D. Fromm M. Takano T. (2000). Complex phenotype of mice lacking occludin, a component of tight junction strands. *Mol. Biol. Cell* 11 4131–4142. 10.1091/mbc.11.12.4131 11102513 PMC15062

[B86] Sakakibara A. Furuse M. Saitou M. Ando-Akatsuka Y. Tsukita S. (1997). Possible involvement of phosphorylation of occludin in tight junction formation. *J. Cell Biol.* 137 1393–1401. 9182670 10.1083/jcb.137.6.1393 PMC2132539

[B87] Sarrazin S. Lamanna W. C. Esko J. D. (2011). Heparan sulfate proteoglycans. *Cold Spring Harb. Perspect. Biol.* 3:a004952. 10.1101/cshperspect.a004952 PMC3119907 21690215

[B88] Segarra M. Aburto M. R. Cop F. Llaó-Cid C. Härtl R. Damm M. (2018). Endothelial Dab1 signaling orchestrates neuro-glia-vessel communication in the central nervous system. *Science* 361:eaao2861. 10.1126/science.aao2861 30139844

[B89] Sladojevic N. Stamatovic S. M. Johnson A. M. Choi J. Hu A. Dithmer S. (2019). Claudin-1-Dependent Destabilization of the Blood-Brain Barrier in Chronic Stroke. *J. Neurosci*. 39 743–757. 10.1523/JNEUROSCI.1432-18.2018 30504279 PMC6343646

[B90] Sohet F. Lin C. Munji R. N. Lee S. Y. Ruderisch N. Soung A. (2015). LSR/angulin-1 is a tricellular tight junction protein involved in blood–brain barrier formation. *J. Cell Biol*. 208 703–711. 10.1083/jcb.201410131 25753034 PMC4362448

[B91] Steed E. Rodrigues N. T. L. Balda M. S. Matter K. (2009). Identification of MarvelD3 as a tight junction-associated transmembrane protein of the occludin family. *BMC Cell Biol*. 10:95. 10.1186/1471-2121-10-95 20028514 PMC2805614

[B92] Stevenson B. R. Siliciano J. D. Mooseker M. S. Goodenough D. A. (1986). Identification of ZO-1: a high molecular weight polypeptide associated with the tight junction (zonula occludens) in a variety of epithelia. *J. Cell Biol*. 103 755–766. 10.1083/jcb.103.3.755 3528172 PMC2114282

[B93] Taddei A. Giampietro C. Conti A. Orsenigo F. Breviario F. Pirazzoli V. (2008). Endothelial adherens junctions control tight junctions by VE-cadherin-mediated upregulation of claudin-5. *Nat. Cell Biol*. 10 923–934. 10.1038/ncb1752 18604199

[B94] Thomsen M. S. Routhe L. J. Moos T. (2017). The vascular basement membrane in the healthy and pathological brain. *J. Cereb. Blood Flow Metab.* 37 3300–3317. 10.1177/0271678x17722436 28753105 PMC5624399

[B95] Tomi M. Hosoya K. J. (2004). Application of magnetically isolated rat retinal vascular endothelial cells for the determination of transporter gene expression levels at the inner blood-retinal barrier. *Neurochem* 91 1244–1248. 10.1111/j.1471-4159.2004.02842.x 15569267

[B96] Tornabene E. Helms H. C. C. Pedersen S. F. Brodin B. (2019). Effects of oxygen-glucose deprivation (OGD) on barrier properties and mRNA transcript levels of selected marker proteins in brain endothelial cells/astrocyte co-cultures. *PLoS One* 14:e0221103. 10.1371/journal.pone.0221103 31425564 PMC6699694

[B97] Tornavaca O. Chia M. Dufton N. Almagro L. O. Conway D. E. Randi A. M. (2015). ZO-1 controls endothelial adherens junctions, cell–cell tension, angiogenesis, and barrier formation. *J. Cell Biol.* 208 821–838. 10.1083/jcb.201404140 25753039 PMC4362456

[B98] Tsukita S. Furuse M. Itoh M. (2001). Multifunctional strands in tight junctions. *Nat. Rev. Mol. Cell Biol.* 2 285–293. 10.1038/35067088 11283726

[B99] Umeda K. Ikenouchi J. Katahira-Tayama S. Furuse K. Sasaki H. Nakayama M. (2006). ZO-1 and ZO-2 independently determine where claudins are polymerized in tight-junction strand formation. *Cell* 126 741–754. 10.1016/j.cell.2006.06.043 16923393

[B100] Vanlandewijck M. He L. Mäe M. A. Andrae J. Ando K. Del Gaudio F. (2018). A molecular atlas of cell types and zonation in the brain vasculature. *Nature* 554 475–480. 10.1038/nature25739 29443965

[B101] Wegmann F. Ebnet K. Du Pasquier L. Vestweber D. Butz S. (2004). Endothelial adhesion molecule ESAM binds directly to the multidomain adaptor MAGI-1 and recruits it to cell contacts. *Exp. Cell Res.* 300 121–133. 10.1016/j.yexcr.2004.07.010 15383320

[B102] Williams D. W. Anastos K. Morgello S. Berman J. W. (2015). JAM-A and ALCAM are therapeutic targets to inhibit diapedesis across the BBB of CD14+CD16+ monocytes in HIV-infected individuals. *J. Leukoc. Biol*. 97 401–412. 25420915 10.1189/jlb.5A0714-347R PMC4304417

[B103] Winger R. C. Koblinski J. E. Kanda T. Ransohoff R. M. Muller W. A. (2014). Rapid remodeling of tight junctions during paracellular diapedesis in a human model of the blood-brain barrier. *J. Immunol*. 193 2427–2437. 10.4049/jimmunol.1400700 25063869 PMC4138548

[B104] Wolburg H. Wolburg-Buchholz K. Kraus J. Rascher-Eggstein G. Liebner S. Hamm S. (2003). Localization of claudin-3 in tight junctions of the blood-brain barrier is selectively lost during experimental autoimmune encephalomyelitis and human glioblastoma multiforme. *Acta Neuropathol.* 105 586–592. 10.1007/s00401-003-0688-z 12734665

[B105] Woodfin A. Reichel C. A. Khandoga A. Corada M. Voisin M. B. Scheiermann C. (2007). JAM-A mediates neutrophil transmigration in a stimulus-specific manner in vivo: evidence for sequential roles for JAM-A and PECAM-1 in neutrophil transmigration. *Blood* 110 1848–1856. 10.1182/blood-2006-09-047431 17505016

[B106] Woodfin A. Voisin M. B. Beyrau M. Colom B. Caille D. Diapouli F. M. (2011). The junctional adhesion molecule-C (JAM-C) regulates polarized neutrophil transendothelial cell migration *in vivo*. *Nat. Immunol.* 12 761–769. 10.1038/ni.2062 21706006 PMC3145149

[B107] Wu C. Ivars F. Anderson P. Hallmann R. Vestweber D. Nilsson P. (2009). Endothelial basement membrane laminin α5 selectively inhibits T lymphocyte extravasation into the brain. *Nat. Med.* 15 519–527. 10.1038/nm.1957 19396173

[B108] Yao Y. Chin Z.-L. Norris E. H. Strickland S. (2014). Astrocytic laminin regulates pericyte differentiation and maintains blood brain barrier integrity. *Nat. Commun.* 5:3413. 10.1038/ncomms4413 24583950 PMC3992931

[B109] Zihni C. Mills C. Matter K. Balda M. S. (2016). Tight junctions: from simple barriers to multifunctional molecular gates. *Nat. Rev. Mol. Cell Biol.* 17 564–580. 10.1038/nrm.2016.80 27353478

